# *Salmonella* in Liquid Eggs and Other Foods in Fukuoka Prefecture, Japan

**DOI:** 10.1155/2013/463095

**Published:** 2013-10-28

**Authors:** Koichi Murakami, Tamie Noda, Daisuke Onozuka, Nobuyuki Sera

**Affiliations:** ^1^Fukuoka Institute of Health and Environmental Sciences, 39 Mukaizano, Dazaifu, Fukuoka 818-0135, Japan; ^2^Chikushi Office for Health, Human Services and Environmental Issues, 3-5-25 Shirakibaru, Onojo, Fukuoka 816-0943, Japan

## Abstract

The study aimed to evaluate the prevalence of *Salmonella* in retail and wholesale foods in Fukuoka Prefecture, Japan. A total of 2,021 samples collected between 1999 and 2010 were tested using a culture method. Samples consisted of liquid eggs (*n* = 30), meat (beef and pork) (*n* = 781), offal (*n* = 69), processed meats (*n* = 2), seafood (*n* = 232), processed seafood (dried fish) (*n* = 76), vegetables (*n* = 481), processed vegetables (*n* = 87), fruits (*n* = 167), and herbs (*n* = 96) from 574 outlets and wholesale agents in 15 areas (five samples were undocumented regarding outlets). Overall, liquid egg showed significantly (*P* < 0.001) higher frequencies of *Salmonella* contamination (13.3%) than beef (1/423, 0.2%) and pork (3/235, 1.3%). *Salmonella enterica* subsp. *enterica* serovar Enteritidis, the most common serovar as a human pathogen, were isolated from two liquid egg samples. No *Salmonella* were isolated from seafood and vegetable-related samples including seed sprouts (*n* = 261). In conclusion, liquid egg is a significant *Salmonella* vehicle, showing a need to continue the vaccination of chickens to prevent *S.* Enteritidis contamination in Japanese eggs. Moreover, further study is needed to evaluate *Salmonella* contamination in seed sprouts with more sampling from retailers there.

## 1. Introduction

The surveillance of *Salmonella* in retail and wholesale foods is essential for the prevention of nontyphoidal salmonellosis, which is one of the most important problems for public health in the world [1], including Japan [2]. *Salmonella* frequently enter the food chain, thereby triggering either sporadic cases or outbreaks of human salmonellosis [1]. Livestock and their products are the most significant foods as vehicles of *Salmonella* [1, 3]. In other foods, such as vegetables [4] and fish [5], *Salmonella* can also be present naturally and cause human salmonellosis. Recent data on the prevalence of *Salmonella* in retail and wholesale foods in Fukuoka Prefecture, Japan, are unavailable; therefore, surveillance is needed for reasons of public health. The aim of the present study is to evaluate the prevalence of *Salmonella* in retail and wholesale foods in Fukuoka Prefecture, Japan.

## 2. Materials and Methods

### 2.1. Sampling Area

A total of 2,021 samples were collected from Fukuoka Prefecture between May 1999 and June 2010 (Table 1). These samples consisted of liquid eggs (*n* = 30), meat (beef and pork) (*n* = 781), offal (*n* = 69), processed meats (*n* = 2), seafood (*n* = 232), processed seafood (dried fish) (*n* = 76), vegetables (*n* = 481), processed vegetables (shop-prepared salad and pickled vegetable) (*n* = 87), fruits (*n* = 167), and herbs (*n* = 96). The samples were collected from 563 retail outlets and 11 wholesale agents (five samples were undocumented regarding outlets) in 15 areas of the prefecture by food hygiene inspectors from 13 health centers that are run by the Fukuoka prefectural government in May, June, July, September, October, November, and December (except for 2010). These inspectors collected foods in accordance with the collection program of the local government (http://www.pref.fukuoka.lg.jp/uploaded/life/58/58308_misc1.pdf, in Japanese, accessed in May 2013). The samples were kept in cool boxes with icepacks, brought to the Fukuoka Institute of Health and Environmental Sciences, and tested within 6 h of collection. Fukuoka Prefecture is located in Kyushu, the main southwestern island of Japan. In 2010, the entire population of the prefecture was 5,071,968 and that of the 15 sampling areas was 2,507,741. 

### 2.2. Meat, Giblets, Offal, and Processed Meat Samples

A total of 852 samples, consisting of beef meat, beef offal, processed beef (roast beef), pork meat, mixed minced beef and pork, unrecorded offal, and unrecorded meat (Tables 1 and 2), were collected from 362 outlets. In 2004, there were, in total, 2,812 meat outlets in these sampling areas (data from the Fukuoka prefectural government).

### 2.3. Liquid Egg Samples

The samples (*n* = 30) were collected from 11 wholesale agents between 2005 and 2010 (Table 1). Two of the 11 wholesale agents were also the only manufacturers of liquid eggs in this sampling area. 

### 2.4. Seafood and Processed Seafood Samples

A total of 308 samples are described in Table 3. 306 of these samples were collected from 149 outlets (Table 1) with the source of two other samples undocumented. In 2004, there were a total of 2,615 seafood outlets in these sampling areas (data from the Fukuoka prefectural government) but no data were available on the number of processed seafood outlets.

### 2.5. Vegetable, Processed Vegetable, Fruit, and Herb Samples (Vegetable-Related Samples)

A total of 831 samples are described in Table 3. Almost all of these samples (828 out of 831) were collected from 205 outlets between 1999 and 2009 (Table 1), with the source of three samples undocumented. In 2006, there were a total of 4,379 vegetable and fruit outlets in these sampling areas (estimated data from the Fukuoka prefectural government).

### 2.6. *Salmonella* Investigation

 Samples were tested for *Salmonella* using a culture method. Samples (25 g) in 225 mL of buffered peptone water (Oxoid Ltd., Basingstoke, UK) were homogenized for one minute in stomachers (Seward Ltd., Sussex, UK) and incubated at 35°C for 18 h. For testing liquid egg, melanterite (64 mg/L) was added to the buffered peptone water. After incubation, 0.5 mL aliquots of the preenriched test liquid egg portions were subcultured in parallel both in a tube with 10 mL of Rappaport-Vassiliadis enrichment broth (Oxoid Ltd.) and in a tube with 10 mL of tetrathionate broth (Oxoid Ltd.) in accordance with a Japanese law, The Food Sanitation Act (http://www.japaneselawtranslation.go.jp/law/detail_main?vm=&id=12, accessed in July 2013). Samples other than liquid egg were tested using the same methods as liquid egg from the beginning of the project in 1999 to September 24, 2006, (*n* = 1,398). From September 25, 2006, to the end of the project in 2010, 0.1 mL aliquots of the preenriched test portions of samples other than liquid egg were added to the Rappaport-Vassiliadis enrichment broth and 1 mL aliquots were also added to the tetrathionate broth (*n* = 1,084), in accordance with a Japanese standard method NIHSJ-01 (National Institute of Health Sciences, http://www.nihs.go.jp/fhm/kennsahou-index.html, accessed in August 2012). These cultures were selectively enriched at 42°C for 18 h. The cultures were then streaked for isolation on differential plating media, using two SMID agar (bioMérieux, Lyon, France) and two XLT4 agar plates (BD Diagnostic Systems, Sparks, Maryland, USA). From October to December 2009, CHROMagar *Salmonella* (Chromagar, Paris, France) and DHL agar (Eiken Chemical Co., Tokyo, Japan) were used instead of SMID and XLT4, in accordance with method NIHSJ-01. The plates were incubated at 35°C for 18–48 h. Suspected *Salmonella* colonies (1–4 colonies/sample) were then profiled biochemically as described by Murakami et al. [6]. Isolates with a profile consistent with *Salmonella* were serotyped using somatic and flagella antisera (Denka Seiken Co., Tokyo, Japan).

### 2.7. Statistical Analysis

Statistical analyses were carried out with two-sample tests for proportions using SAS Software, version 9.1.3 (SAS Institute Inc., Cary, NC, USA) with any test showing *P* < 0.01 being considered as statistically significant.

## 3. Results and Discussion

Table 1 shows the prevalence of *Salmonella* in retail and wholesale samples of meat, offal, and processed meats. Samples of beef meat (1/423), cattle offal, (1/68), pork meat (3/235), and mixed minced beef and pork (1/58) were contaminated with *Salmonella* (Table 2). *Salmonella* were also isolated from four of 30 liquid egg samples, showing that the samples harbored significantly more* Salmonella* (*P* < 0.001) than the other groups of samples in Table 1 and both beef pork samples in Table 2. The *Salmonella* consisted of *Salmonella enterica* subsp. *enterica* serovar (*S.*) Enteritidis (*n* = 2) in 2006 and 2009, *S*. Montevideo (*n* = 1) in 2008, and *S*. Braenderup (*n* = 1) in 2010. However, no *Salmonella* were isolated from seafood, processed seafood, or vegetable-related samples (Table 1).

In this study we have shown that liquid egg samples showed significantly higher frequencies of *Salmonella* contamination (13.3%) than beef (0.2%) and pork (1.3%) samples whereas seafood, processed seafood, vegetable, processed vegetable, fruit, and herb samples harbored no *Salmonella*. The samples tested, except for the liquid egg and dried fish samples, might possibly be representative of the foods in the sampling areas because the proportion of outlets tested in the long term study, 12.9% (362/2,812) for meat outlets and 5.7% (149/2,615) for seafood outlets, was higher than 4.7% (205/4,379) for vegetable and fruit outlets that had the lowest level of coverage. 

These results on contamination levels in retail and wholesale food samples have been compared with other studies. The results for liquid egg (13.3%) were comparable to those from our previous study (18.6%) in 1995–1998 [6]. Namimatsu et al. [7] reported that 30.2% of 53 Japanese liquid egg samples were contaminated with *Salmonella *(sampling years were undocumented). Ohtsuka et al. [8] also reported a high frequency of *Salmonella* in Japanese liquid eggs (78.6%–100% from 24–28 samples) from four manufacturers in 2003. The chicken egg is one of the most important infection sources of *S*. Enteritidis, the most common serovar as a human pathogen [1]. Vaccinations are carried out to prevent *S*. Enteritidis-contamination in Japanese eggs [9]. According to Esaki et al. [10], vaccination played an important role in reducing the *Salmonella *contamination levels in eggs from 0.03% (1990–1992) to 0.003% (2010-2011). Our results show the need for further programs of vaccination.

The results for pork (1.3%) were also comparable to those from other studies: 0% in Fukuoka, Japan, in 1995–1998 [6], 3.8% in a nationwide study in Japan on ground pork from 2000 to 2008 [11], 1.9% in the United Kingdom from 2003 to 2005 [12], and 3.2% in Japan (sampling years were undocumented) [13]. The contamination frequency for *Salmonella* in beef (0.2%) was comparable to those in a nationwide study in Japan from 2000–2008 (1.5% in ground beef) [11], the UK (1.1%, 18/1514) in 2003 to 2005 [12], and the USA (1.9%) in 1999–2000 [14]. As many other reports have evaluated contamination in minced beef [15], these values are not comparable with those from the present study. Seafood *Salmonella* contamination has been reported in China (20.8%) in 2005 [16] while no *Salmonella* were detected from seafood in this study. Of course, when comparing our results with previous studies, we must take into account several factors, such as differences in sampling procedures, origin, age of the animals at slaughter, and level of sanitation [17, 18]. Therefore, we are unable to compare them directly. Despite this, we believe that the frequency of contaminated beef (0.2%) and pork (1.3%) in the present study cannot be interpreted as a high frequency. 

Vegetables, especially seed sprouts, have often harbored *Salmonella* [19, 20], whereas no *Salmonella* were isolated from vegetables including seed sprouts (*n* = 261) in this study. However, other studies have shown *Salmonella* contamination in seed sprouts: Fahey et al. [21] tested seed sprout samples and only 24 (0.75%) of the 3191 samples (in 2001) gave a positive response for *Escherichia coli* or *Salmonella*. Another study in Japan has also shown a low frequency of *Salmonella* in seed sprouts: only seven samples (0.1%) from 4,848 seed sprout samples harbored *Salmonella* between 1998 and 2008 [11]. Therefore, further study is required to evaluate *Salmonella* contamination in seed sprouts by collecting more samples in Fukuoka Prefecture, Japan.

## 4. Conclusion

Liquid egg is an important *Salmonella* vehicle, showing significantly higher frequencies of contamination (13.3%) compared with pork (1.3%) and beef (0.2%) in the present study (*P* < 0.001). Seafood, processed seafood, vegetable, processed vegetable, fruit, and herb samples harbored no *Salmonella* whereas further study is needed to evaluate *Salmonella* contamination in seed sprouts with a further collection of samples in Fukuoka Prefecture, Japan. Our results also show the need to continue the vaccination of chickens to prevent *S*. Enteritidis contamination in Japanese eggs.

## Figures and Tables

**Table 1:** Prevalence of *Salmonella* in retail food samples from Fukuoka Prefecture.

Samples	Number of samples tested by year												
	1999	2000	2001	2002	2003	2004	2005	2006	2007	2008	2009	2010	Total
Meat, offal, and processed meat	65	60	87 (1)^†^	73 (2)	73	76	81 (1)	76	83 (1)	78 (1)	78	22	852 (6)^§^
Liquid eggs	0	0	0	0	0	0	5	5 (1)^‡^	5	5 (1)	5 (1)	5 (1)	30 (4)**
Seafood and processed seafood*	55	30	30	30	25	25	20	20	20	20	23	10	308
Vegetables, processed vegetables, fruits, and herbs*	70	90	80	80	70	70	75	75	75	75	71	0	831

	190	180	197	183	168	171	181	176	183	178	177	37	2,021

*No *Salmonella* were isolated from seafood, processed seafood, vegetables, processed vegetables, fruit, and herbs.

^†^Number of S*almonella-*positive samples.

^‡^In liquid eggs, *Salmonella enterica* subsp. *enterica* serovar (*S*.) Enteritidis were isolated in 2006 and 2009. *S*. Montevideo and *S*. Braenderup were also isolated in 2008 and 2010, respectively.

^§^Meat, offal, and processed meat samples harbored significantly more* Salmonella *than the seafood and processed seafood samples (*P* < 0.001); there was no difference in the incidence of *Salmonella* between the meat, offal, and processed meat samples and vegetable, processed vegetable, fruit, and herb samples (*P* = 0.503).

**Liquid egg samples harbored significantly more *Salmonella* than the other three groups of samples (*P* < 0.001).

**Table 2:** *Salmonella*-positive rates among meat, offal, and processed meat samples.

Meat and offal	Number of samples	Number of *Salmonella*-positives (%)	Serovars isolated	*P* values obtained from two-sample tests for proportions between each sample and liquid egg sample
Beef meat	423	1 (0.2%)	*S. *Infantis* (in 2008)	<0.001
Cattle offal	68	1 (1.5%)	*S.* Corvallis and O-untypeable (in 2007)	0.014
Processed beef (roast beef)	2	0 (0.0%)		0.581
Pork meat	235	3 (1.3%)	*S. *Infantis (in 2001 and 2002), O-untypeable (in 2002)	<0.001
Mixed minced beef and pork	58	1 (1.7%)	*S.* Typhimurium (in 2005)	0.026
Unrecorded offal	1	0 (0.0%)		Number of samples insufficient for analysis
Unrecorded meat	65	0 (0.0%)		0.003

Total	852	6 (0.7%)		

*S., *Salmonella enterica* subsp. *enterica *serovar.

**Table:** (a)

Seafood and processed seafood samples	Details	Number of samples
Beloniformes (*n* = 2)	Saury pike	2
Clupeiformes (*n* = 1)	Sardine	1
Gadiformes (*n* = 1)	Cod	1
Mugiliformes (*n* = 2)	Bora	2
Myliobatiformes (*n* = 1)	Whip ray	1

Perciformes (*n* = 185)	Amberjack	33
	Big-eyed tuna	1
	Common sea bass	7
	Dorado	3
	Hair tail	2
	Horse mackerel	12
	Mackerel	7
	Redfish	1
	Sea bream	73
	Skipjack (bonito)	2
	Striped horse mackerel	1
	Striped pigfish	2
	Swordfish	1
	Tilefish	1
	Tuna	4
	White croaker	1
	Yellowtail	34

Pleuronectiformes (*n* = 14)	Bastard halibut	12
	Flat fish	2

Salmoniformes (*n* = 11)	Atlantic salmon	6
	Coho salmon	2
	Salmon	3

Scorpaeniformes (*n* = 2)	Black cod	1
	Flathead	1

Other seafood (*n* = 13)	Scallop	1
	Squid	8
	Shrimp	4

Processed seafood (*n* = 76)	Dried fish	76

	Total	308

**Table:** (b)

Vegetable, processed vegetable, fruit, and herb samples	Details	Number of samples
Bulb and stem vegetables (*n* = 5)	Celery	2
	Leek	1
	Welsh onion	2

Leafy and salad vegetables (*n* = 201)	Cabbage	16
	Chinese cabbage	39
	Green lettuce	1
	Leaf lettuce	11
	Leaf of *daikon* (white radish)	1
	Lettuce	101
	*Nozawana* (turnip greens)	1
	Potherb mustard	3
	Red leaf lettuce	5
	Spinach	23

Root and tuberous vegetables (*n* = 14)	Carrot	1
	*Daikon* (white radish)	10
	Turnip	3

Seed sprouts (*n* = 261)	Alfalfa	5
	Bean sprout	122
	Broccoli sprout	7
	Japanese radish sprout	126
	Mustard sprout	1

Processed vegetables (*n* = 87)	Shop-prepared salad	74
	Pickled vegetable	13

Fruits (*n* = 167)	Cucumber	105
	Eggplant	8
	Tomato	54

Herbs (*n* = 96)	Japanese honeywort	93
	*Myoga *(ginger)	1
	Parsley	2

	Total	831
